# Investigation of Associations between *NR1D1, RORA* and *RORB* Genes and Bipolar Disorder

**DOI:** 10.1371/journal.pone.0121245

**Published:** 2015-03-19

**Authors:** Yin-Chieh Lai, Chung-Feng Kao, Mong-Liang Lu, Hsi-Chung Chen, Po-Yu Chen, Chien-Hsiun Chen, Winston W. Shen, Jer-Yuarn Wu, Ru-Band Lu, Po-Hsiu Kuo

**Affiliations:** 1 Department of Public Health & Institute of Epidemiology and Preventive Medicine, College of Public Health, National Taiwan University, Taipei, Taiwan; 2 Department of Psychiatry, Taipei Medical University-Wan Fang Medical Center, Taipei, Taiwan; 3 Department of Psychiatry & Center of Sleep Disorders, National Taiwan University Hospital, Taipei, Taiwan; 4 Department of Psychiatry, Taipei City Psychiatric Center, Taipei City Hospital, Taipei, Taiwan; 5 Institute of Biomedical Sciences, Academia Sinica, Taipei, Taiwan; 6 Department of Psychiatry, National Cheng Kung University Hospital, Tainan, Taiwan; 7 Research Center for Genes, Environment and Human Health, National Taiwan University, Taipei, Taiwan; Johns Hopkins Bloomberg School of Public Health, UNITED STATES

## Abstract

Several genes that are involved in the regulation of circadian rhythms are implicated in the susceptibility to bipolar disorder (BD). The current study aimed to investigate the relationships between genetic variants in *NR1D1 RORA*, and *RORB* genes and BD in the Han Chinese population. We conducted a case-control genetic association study with two samples of BD patients and healthy controls. Sample I consisted of 280 BD patients and 200 controls. Sample II consisted of 448 BD patients and 1770 healthy controls. 27 single nucleotide polymorphisms in the *NR1D1, RORA*, and *RORB* genes were genotyped using GoldenGate VeraCode assays in sample I, and 492 markers in the three genes were genotyped using Affymetrix Genome-Wide CHB Array in sample II. Single marker and gene-based association analyses were performed using PLINK. A combined p-value for the joining effects of all markers within a gene was calculated using the rank truncated product method. Multifactor dimensionality reduction (MDR) method was also applied to test gene-gene interactions in sample I. All markers were in Hardy-Weinberg equilibrium (P>0.001). In sample I, the associations with BD were observed for rs4774388 in *RORA* (OR = 1.53, empirical p-value, P = 0.024), and rs1327836 in *RORB* (OR = 1.75, P = 0.003). In Sample II, there were 45 SNPs showed associations with BD, and the most significant marker in *RORA* was rs11639084 (OR = 0.69, P = 0.002), and in *RORB* was rs17611535 (OR = 3.15, P = 0.027). A combined p-value of 1.6×10−6, 0.7, and 1.0 was obtained for *RORA, RORB* and *NR1D1*, respectively, indicting a strong association for *RORA* with the risk of developing BD. A four way interaction was found among markers in *NR1D1, RORA*, and *RORB* with the testing accuracy 53.25% and a cross-validation consistency of 8 out of 10. In sample II, 45 markers had empirical p-values less than 0.05. The most significant markers in *RORA* and *RORB* genes were rs11639084 (OR = 0.69, P = 0.002), and rs17611535 (OR = 3.15, P = 0.027), respectively. Gene-based association was significant for *RORA* gene (P = 0.0007). Our results support for the involvement of *RORs* genes in the risk of developing BD. Investigation of the functional properties of genes in the circadian pathway may further enhance our understanding about the pathogenesis of bipolar illness.

## Introduction

Bipolar disorder (BD) that is known as manic-depressive illness, affects approximately 1% of the general population [1] with high heritability [2]. BD patients frequently demonstrate biological rhythm-related symptoms, including diurnal variation of mood and sleep disturbances (e.g., a decreased need for sleep during manic episode, and insomnia or hypersomnia during depressive episode). Moreover, sleep disturbances aggravate emotional dysregulation [3] and are reported to predict relapse of mood episode [4]. BD patients are also characterized by instable social rhythms, such as early awakening, lower activity during depressive episode or higher activity during manic episode. Together with these observations among BD patients, abnormality in circadian function has been postulated to play important roles in the pathophysiology of BD [5].

The circadian system is critical for proper regulation of behavioral and physiological rhythms in human, which mastered by the suprachiasmatic nucleus (SCN) in the hypothalamus. It is regulated by two major transcriptional feedback loops [6]. One positive major transcriptional activator of circadian system is a dimer consisted of products encoded by two genes, the circadian locomotor output cycles kaput (*CLOCK*) and brain and muscle ARNT-like protein 1 (*ARNTL*, also known as *BMAL1*). The other negative feedback loop is regulated by the period (PER) and cryptochrome (CRY) proteins that are translated in the cytoplasm to repress the actions of CLOCK/ARNTL [7]. Recently, an additional negative feedback loop is reported to involve with the two orphan nuclear receptors [8]: nuclear receptor subfamily 1, group D member 1 (*NR1D1*, also known as *rev-erb*-*α*) and retinoic acid–related orphan receptor-A (*RORA*). The NR1D1 protein enters the nucleus to repress transcription of *CLOCK/ARNTL*, while RORA protein has opposite transcriptional function to activate *ARNTL*. Thus, the transcription of *ARNTL* is the result of competition between *NR1D1* and *RORA* [9].

In the past, several circadian genes have reported to be associated with shorter total sleep time [10], insomnia [11] and daytime fatigue [12] in clinical patients with mood disorders. However, relatively few studies exist to examine the associations of *ROR* gene family in patients with BD. The *ROR* family contains 3 different genes: *RORA, RORB*, and *RORC*. Among them, only *RORC* does not express in the SCN [13]. The *RORA* or *RORB* deficient mice exhibit aberrant circadian behaviors, whereas no abnormalities in circadian behaviors have been noticed in *RORC* ^−/−^ mice [14, 15]. In human, few studies ever examined the associations between genetic variants in *NR1D1, RORA*, and *RORB* genes and BD, which we summarize their results in Table 1. For *NR1D1*, both positive [16] and negative [17] findings are reported in Caucasian populations. One study conducted in Japanese reported no association for *NR1D1* with BD at single marker level, but shows association in one haplotype in the *NR1D1* gene with onset age of manic episode [18]. Moreover, there are three studies examined genes in the *ROR* family. McGrath et al. [19] reported associations for *RORB* but not *RORA* with BD subtype I. Using a sample with around 500 cases and 500 controls, Mansour et al. [20] also reported nominal significance for *RORB* with BD subtype I. On the contrary, Soria et al. [21] found significant associations for *RORA* with BD. With the few studies conducted so far without consistent findings, we aimed to examine the roles of *NR1D1, RORA*, and *RORB* genes in the pathogenesis of BD.

**Table 1:** Association studies of *NR1D1, RORA*, and *RORB* genes in bipolar disorder.

Genes	Study	Study design	Race	Subjects	No. of SNPs	Results	Results description	OR	p-value
*NR1D1*	Kishi et al., [18]	case control	Japanese	147 BD, (93 BD-I, 54 BD-II), 322 MDD, 360 controls	3	−	No significant in BD		
	Shi et al., [22]	family based	Caucasian	114 BD families	4	−	No significant in BD		
	Kripke et al., [16]	case control family based	Caucasian	444 BD families (80% BD-I), 130 unrelated BD, 149 MDD families, 360 sleep clinic samples	11	+	rs2314339^†^, rs2071427 and rs2269457 were associated with BD in BD family	0.610.750.79	0.0005^†^, 0.0019, 0.0292
	Severino et al., [17]	case control	Sardinian	300 BD (181 BD-I, 74 schizoaffective bipolar, 45 BD-II), 300 controls	14	−	no significant in 14 single markers with BD, but in one haplotype of rs12941497 and rs939347 was associated with onset age in BD	1.68	0.025
	Soria et al., [21]	case control	Spanish	199 BD, 335 MDD, 440 controls	6	−	No significant in BD rs2071427 was associated with MDD (OR = 1.80, P = 0.039)		
*RORA*	McGrath et al., [19]	case control family based	Caucasian	152 BD-I, 140 controls, 153 trios	332	−	No significant in BD		
	Soria et al., [21]	case control	Spanish	199 BD, 335 MDD 440 controls	24	+	rs4774370 was associated with BD	2.26	0.031
*RORB*	Mansour et al., [20]	case control	Caucasian	523 BD-I, 477 controls	46	+	rs10491929, rs10217594, and rs17691363 were associated with BD-I		0.023, 0.026, 0.035
	McGrath et al., [19]	case control family based	Caucasian	152 BD-I, 140 controls, 153 trios	44	+	rs1157358^†^, rs7022435^†^, rs3750420^†^, rs3903529^†^ were associated with BD-I no significant in trios		<0.0001^†^,<0.0001^†^,<0.0001^†^ ^,^ <0.0001^†^

No. of SNPs: number of genotype SNPs in the study

^†^Statistically significant after correction for multiple testing

SNP: single-nucleotide polymorphism; OR: odds ratio

The current study attempted to examine the associations between genetic variants in the *NR1D1, RORA*, and *RORB* genes with BD using a culturally and ethnically more homogeneous sample in the Han Chinese population. We also reasoned that genes in the same feedback loop might interact with each other due to the complex nature resides in the biological functions of the circadian system. Thus, we intended to examine the interaction effects among genetic variants in *NR1D1, RORA* and *RORB* genes using multifactor dimension reduction (MDR) approach.

## Materials and Methods

We conducted a case control genetic association study for the *NR1D1, RORA* and *RORB* genes with BD. Two samples were tested for these three genes. Sample I was genotyped in early stage based on selection of candidate genetic variants using a customized array chip, and Sample II was genotyped in 2013 using a genome-wide association (GWA) chip in which we analyzed markers in the three genes.

### Participants

Clinical patients who met the diagnostic criteria of bipolar I disorder (BD-I) or bipolar II disorder (BD-II) according to the Diagnostic and Statistical Manual of Mental Disorders-IV (DSM-IV) were consecutively referred by psychiatrists in Taiwan from 2008 to 2012. Index probands whose age between 18 and 70 years and their biological parents were Han Chinese were recruited. Patients who diagnosed with schizophrenia, schizoaffective or substance-induced mood disorders and whose diagnoses were changed during the data collection period were excluded. Healthy controls were recruited in the community and were screened for mood disturbances and other major psychiatric disorders on the basis of structured interviews. More details of sample recruitment please refer to Tsai et al., [22]. The present study was approved by the Institutional Review Board of National Taiwan University Hospital, National Cheng Kung University Hospital, and Taipei City Hospital. All clinical participants were evaluated by psychiatrists in the participating hospitals for their capacity to sign inform consent. Only patients who had the ability to consent on participation of this study were referred and all our participants provided written informed consent.

Participants were interviewed by well-trained interviewers using the Composite International Diagnostic Interview (CIDI) [23] and the Chinese version of the modified Schedule of Affective Disorder and Schizophrenia-Lifetime (SADS-L) to collect data on demographic and clinical features, and information on major psychiatric disorders (details please refer to Tsai et al., [22]. In addition, blood samples were taken to extract DNA for each individual. Subjects who provided blood samples and completed the interview were included in the case-control association study. For Sample I, we genotyped 280 BD patients (200 BD-I, 80 BD-II), and 200 healthy controls. For Sample II, we genotyped 448 BD patients (262 BD-I, 186 BD-II) and 1770 healthy controls. Control samples in Sample II were community subjects drawn from the Han Chinese Cell and Genome Bank in Taiwan [24]. Subjects were screened for major disease history (including major mental illnesses), however, no detailed interview data were provided.

### Experimental designs and quality control

DNA was extracted from peripheral white blood cells using commercial kit and stored at −80°C. We diluted DNA samples to 50ng/μL; concentration and quality were checked using Quant-iT PicoGreen dsDNA Reagent and Kits. After DNA extraction, we then selected markers in the *NR1D1, RORA*, and *RORB* genes for genotyping.

The selection of single nucleotide polymorphism (SNPs) for genotyping in Sample I was based on two criteria. First, we chose SNPs that showed genetic associations with BD in the literature (see the summary Table 1). Second, we searched coding SNPs in the three genes from NCBI dbSNP database (BUILD 37.3). Markers that have been genotyped in HapMap project (http://hapmap.ncbi.nlm.nih.gov/), with minor allele frequency greater than 0.01 in the CHB (Han Chinese in Beijing, China) samples were considered in the present study. We intended to genotype in total 27 SNPs according to these two selection processes. We used customized Illumina GoldenGate Genotyping Assay with VeraCode Technology to genotype the selected 27 SNPs at the National Taiwan University Center for Biotechnology. Genotyping data showed that two markers (rs1568717 and rs17691363) had low (<5%) minor allele frequency (MAF). Therefore, we discarded these two SNPs in the following analysis. For the remaining 25 SNPs, the call rate was high, ranging from 95.8% to 99.6%, and had p-value greater than 0.05 in the Hardy-Weinberg equilibrium (HWE) test. Three individuals had missing data for all SNPs and were excluded from analysis, including two BD-I and one BD-II patients.

For Sample II, individual genotyping was conducted using the Axiom Genome-Wide CHB Array with 628,132 SNPs in the Genomics Research Center in Academia Sinica. This array is specifically designed for CHB populations with the highest coverage rate of common variants for the Han Chinese populations. Using 20kb upstream and downstream of the gene boundaries, 492 SNPs were mapped into *NR1D1, RORA*, and *RORB* protein-coding genes. For Sample II, 18 individuals (3 BD-I and 15 controls) had call rate less than 95% and were dropped from the analysis. To evaluate kinship relationship among samples, we calculated inbreeding coefficient and identity by state (IBS) to eliminate samples with a strong kinship relationship using PLINK (Purcell, http://pngu.mgh.harvard.edu/∼purcell/plink/) [25]. Subjects with inbreeding coefficients outside the mean ±3×standard deviation or subjects not in the same cluster with others using the IBS distance value were removed. As a result, 58 individuals (48 controls, 5 BD-I and 5 BD-II) were discarded. Additionally, population stratification was evaluated by multidimensional scaling analysis and 8 controls were removed from analysis. In total, 2134 individuals (254 BD-I, 181 BD-II, and 1699 controls) were retained after quality control procedures.

We then performed quality controls for the 492 markers. We removed SNPs with poor amplification, HWE test p-values less than 0.001, genotype missing rate greater than 5%, MAF smaller than 0.05, and bad calling in clustering. We also compared B allele among two groups (i.e., cases versus whole sample, controls versus whole sample) to remove markers whose difference of B allele frequency greater than 2%. As a result, 429 markers were retained in the following analysis. There were 10 markers genotyped in both samples. We checked the MAF of them in the two samples.

### Statistical analysis

Because our patients group consisted of BD-I and BD-II, we first conducted exploratory analysis for the two subtypes to see whether MAF distributions are different in the two subtypes of BD. There were no significant differences between BD-I and BD-II, thus, the two subgroups were combined together into the overall BD group. We performed genetic association analyses using PLINK at single marker and gene-based levels, and odds ratios (OR) were estimated. For single marker analysis, we tested additive, dominant and recessive genetic models, and used a maximum test for associations [26]. Empirical p-values were generated using the max (T) permutation approach for pointwise estimates (EMP1). For gene-based analysis, SNPs were assigned to a gene if they located within the gene region. We adopted the default setting in PLINK to retain SNPs with linkage disequilibrium (LD) lower than 0.5, and had p-value < 0.1. The significance level of each gene was obtained through 10,000 permutations. Because the three genes were chosen with a higher priori to be associated with BD, we also used the rank truncated product method (TPM) to take the issue of multiple testing into account [27]. It is assumed that a set of weakly significant markers may altogether contribute to the risk of developing diseases. Thus, rather than doing association testing for each individual marker, the TPM calculates a combined p-value for the joining effects of all single markers within a gene. A fixed threshold τ was chosen to dichotomize SNPs into significant and non-significant marker-set within a gene. We set τ = 0.05 in corresponding to a nominal p-value of 0.05 [27]. All statistical tests were two-sided and the significance level was set at 0.05. Because there were few SNPs genotyped in sample I for each gene, we calculated gene-based associations using sample II.

We also performed the MDR analysis [28] to detect gene-gene interactions among 27 SNPs genotyped in Sample I. While many traditional methods are failed or with limited power to characterize epistasis effects, especially with small sample size [29], the MDR analysis can examine gene-gene interaction without main effects. The MDR approach is a model-free and non-parametrical method to identify high dimensional interactions among multiple factors assuming that combinations of genetic factors provide the most information in the classification of high versus low risk groups. It is suggested to be a useful method to identify gene-gene interactions in high dimensional data [30]. There are three steps in conducting MDR analysis. First, we divided samples into training and testing datasets to perform ten-fold cross-validation to avoid the over-fitting problem in the model. Second, a set of *n* genetic markers were selected by the models to form all possible genotypic combinations. For example, for two loci with three genotypes each, there are nine possible two-locus combinations. The ratio of cases/controls within each combination can then be calculated. Finally, a high or low risk group is assigned for each genotypic combination based on the comparison of case/control ratio. If the case/control ratio of a multifactor combination is higher than that in the sample population, the combination is assigned a high risk group, and *vice versa*. The best marker combination is selected according to the testing-balanced accuracy and cross-validation consistency.

## Results

In Sample I, there was 48.6% and 31.0% males in BD patients (age 34.8 ± 11.5 years), and controls (age 47.3 ± 8.7 years), respectively. In Sample II, there was 45.5% and 46.4% males in BD patients (age 36.4 ± 12.5 years), and controls (age 46.2 ± 14.7 years). The characteristics of demographic variables in the two samples were shown in Table 2.

**Table 2:** Characteristics of demographic variables in the two samples.

	Sample I		Sample II	
	BD (N = 280)	Controls (N = 200)	BD (N = 435)	Controls (N = 1699)
	Mean (SD)	Mean (SD)	Mean (SD)	Mean (SD)
Age at interview, years	34.80 (11.5)	47.30 (8.7)	36.40 (12.5)	46.20 (14.7)
Education, years	13.15 (5.66)	10.92 (4.38)	11.74 (4.78)	
	N (%)	N (%)	N (%)	N (%)
Gender				
Male	137 (48.6)	62 (31.0)	198 (45.5)	788 (46.4)
Marital status				
Married	95 (34.1)	118 (59.0)	100 (23.0)	
Separated, divorced, widowed	52 (18.3)	30 (14.8)	60 (13.9)	
Never married	133 (47.6)	52 (26.2)	275 (63.1)	

Table 3 displays the characteristics of associated markers in the two samples. All SNPs were under the HWE (P>0.001) and the MAF was greater than 0.05. In sample I, one marker each in the *RORA* and *RORB* genes showed associations with BD (P<0.05). The minor allele of rs4774388 in *RORA* had an increased risk for BD (OR = 1.53, P = 0.024), and marker rs1327836 in *RORB* revealed association with BD (OR = 1.75, P = 0.003) in dominant model. In Sample II, there were 45 SNPs (43 SNPs in *RORA* and 2 SNPs in *RORB*) showed associations with BD. The most significant marker in each gene was rs11639084 in *RORA* (OR = 0.69, P = 0.002) and rs17611535 in *RORB* (OR = 3.15, P = 0.027). One marker reported in sample I (rs4774388) did not show association with BD in Sample II. With a joint analysis to combine results from the two samples using Comprehensive Meta-Analysis Version 2 software [31], the overall p-value of this marker with BD become non-significant (P = 0.19).

**Table 3:** Single marker associations with bipolar disorders (P<0.05).

				MAF			BD vs. Control	
Gene	Ch	SNP	Position	BD	Control	Model	OR^a^	EMP
*RORA*	15	rs8041381	60862962	0.133	0.108	ADD	1.31	0.022
		rs11632600	60873670	0.224	0.207	REC	1.90	0.010
		rs75981965	60918491	0.056	0.070	DOM	0.68	0.028
		rs339998	60947763	0.435	0.409	DOM	1.30	0.030
		rs2553236	60986572	0.284	0.306	REC	0.63	0.035
		rs13329238	60998847	0.227	0.210	REC	1.75	0.030
		rs17237353	61021047	0.145	0.178	DOM	0.72	0.008
		rs1020729	61045072	0.395	0.433	ADD	0.85	0.043
		rs1020730	61048578	0.208	0.244	ADD	0.78	0.014
		rs12900122	61055411	0.155	0.186	DOM	0.74	0.016
		rs8025689	61059261	0.127	0.165	ADD	0.72	0.007
		rs9302215	61060367	0.307	0.344	ADD	0.84	0.044
		rs1482057	61064751	0.169	0.139	ALL	1.27	0.023
		rs11639084	61066516	0.128	0.169	ADD	0.69	0.002
		rs12594188	61067805	0.222	0.260	ALL	0.81	0.025
		rs10519067	61068347	0.108	0.134	ADD	0.74	0.021
		rs11071557	61068954	0.145	0.186	ADD	0.71	0.003
		rs11071558	61069421	0.147	0.187	ADD	0.72	0.005
		rs922781	61070344	0.252	0.225	DOM	1.29	0.021
		rs1963497	61071791	0.111	0.135	ADD	0.77	0.044
		rs17270446	61073802	0.139	0.159	REC	0.38	0.040
		rs877228	61076591	0.387	0.406	REC	0.71	0.034
		rs16943117	61078836	0.167	0.201	ADD	0.78	0.017
		rs12915776	61079377	0.154	0.193	ADD	0.74	0.004
		rs6494227	61106118	0.087	0.114	DOM	0.71	0.019
		rs79610262	61106460	0.086	0.113	DOM	0.70	0.017
		rs78507043	61114212	0.449	0.489	DOM	0.75	0.035
		rs16943172	61117865	0.267	0.301	DOM	0.78	0.026
		rs75084363	61145871	0.141	0.142	REC	2.84	0.005
		rs8041466	61155328	0.227	0.225	REC	1.76	0.021
		rs11631432	61174348	0.422	0.437	REC	0.70	0.026
		rs2414686	61194350	0.505	0.469	DOM	1.38	0.014
		rs12910281	61195749	0.485	0.452	REC	1.38	0.021
		rs2899664	61200750	0.246	0.208	ALL	1.25	0.016
		rs11855147	61202300	0.075	0.103	ADD	0.66	0.006
		rs79995443	61224299	0.084	0.106	DOM	0.69	0.015
		rs72752802	61270307	0.370	0.391	REC	0.71	0.034
		rs74687025	61324825	0.133	0.151	REC	0.27	0.018
		rs4775350	61333253	0.202	0.228	REC	0.43	0.007
		rs782908	61378610	0.148	0.148	REC	0.20	0.030
		rs76824799	61385004	0.103	0.135	ADD	0.74	0.014
		rs4774388*	61466998	0.383/(0.384^†^)	0.378/(0.348^†^)	DOM	1.14/(1.53^†^)	0.366/(0.024^†^)
		rs34720147	61498706	0.449	0.408	ALL	1.19	0.029
		rs11631786	61512161	0.158	0.189	ALL	0.80	0.041
*RORB*	9	rs17611535	77158236	0.089	0.085	REC	3.15	0.027
		rs1327836*	77292415	NA/(0.373^†^)	NA/(0.308^†^)	DOM	NA/(1.75^†^)	NA/(0.003^†^)
		rs499922	77312690	0.215	0.182	ALL	1.22	0.042

^a^ Regression models were adjusted for age at interview and sex

A combined p value using the rank truncated product method was 1.6×10^–6^ for *RORA* (threshold τ = 0.05); 0.007 for *RORB* (if use a loose threshold *τ* = 0.1)

* SNPs in Sample I with empirical p-value<0.05

^†^ MAF, OR, and p-value in Sample I

SNP: single-nucleotide polymorphism; Ch: chromosome; MAF: Minor Allele Frequency OR: odds ratio; EMP: Empirical p-values were generated using the max (T) permutation approach for pointwise estimates; DOM: dominant model; REC: recessive model; ALL: allelic model; ADD: additive model; NA: not genotyped in GWAS chip

For gene-based analysis, there were 47 markers in *RORA* and 3 in *RORB* with p-values less than the threshold of 0.05 and were truncated into the significant marker-set, while the remaining 392 (346 in *RORA*, 43 in *RORB*, 3 in *NR1D1*) SNPs were in the ‘non-significant’ marker-set. TPM analysis yielded a p-value of 1.6×10^−6^, 0.7, and 1.0 for *RORA, RORB* and *NR1D1*, respectively. Notably, the association of *RORB* with BD (combined p-value = 0.007) was significant using a loose threshold of τ = 0.1. These results indicated strong association of *RORA*, weak association of *RORB*, and no association of *NR1D1* with BD for the main effects. For the MDR analysis, the two- to four-way gene-gene interaction models are listed in Table 4. Consistent with the single marker analysis, the rs1327836 in the *RORB* gene had the highest testing-balanced accuracy among the 25 SNPs using sample I data. A four-way gene-gene interaction among markers rs2071427 (*NR1D1*), rs4774388 (*RORA*), rs3750420 (*RORB*), and rs11144047 (*RORB*) showed the highest training-balanced accuracy (70.16%), testing-balanced accuracy (53.25%) and cross validation consistency (8/10).

**Table 4:** Summaries of multifactor dimension reduction gene-gene interaction results in sample I.

Gene1	SNP1	Gene2	SNP2	Gene3	SNP3	Gene4	SNP4	Training Bal. Acc. (%)	Testing Bal. Acc. (%)	Cross validation consistency
*RORB*	rs1327836							0.5778	0.5200	7/10
*RORB*	rs1327836	*NR1D1*	rs12941497					0.5971	0.5239	3/10
*RORB*	rs1327836	*RORB*	rs3750420	*RORA*	rs809736			0.6355	0.4807	2/10
*RORB*	rs3750420	*RORB*	rs11144047	*RORA*	rs4774388	*NR1D1*	rs2071427	0.7016	0.5325	8/10

Training Bal. Acc.: training-balanced accuracy

Testing Bal. Acc.: testing-balanced accuracy

SNP: single-nucleotide polymorphism

## Discussion

Disruption of the circadian clock has been implicated in the etiology of BD in prior behavioral, genetic and biochemical studies. Few genetic data directly addressed the roles of *NR1D* and *ROR* gene families in BD, and none examined their interaction effects. We found that several genetic variants in the *RORA* and *RORB* genes were associated with BD. In addition, important four-way gene-gene interactions were detected among *NR1D1, RORA*, and *RORB* genes. Our findings support the potential roles of these circadian genes in the pathogenesis of BD.

In Table 1, we summarized previous findings of the three genes, and inconsistent results are seen in the *NR1D1* and *RORA* genes with both positive and negative findings. Most of the study samples consisted of a mixture of BD subtype I and II diagnoses (the proportion of BD subtype I ranged from 63–80%). It is unlikely that the mixture of diagnoses can fully explain the inconsistent association findings. One possible explanation is that none of the individual genetic association studies genotyped genetic variants in full coverage for these candidate genes. Thus, negative findings may result from non-genotyped variants. We also noticed that the only significant association in the *NR1D1* gene is from a family-based design [16], while all other case-control studies fail to show the main effect of *NR1D1* with BD. Lastly, we are interested in exploring interaction effects between *NR1D1* and *RORs* gene family as they are in the same transcriptional feedback loop in circadian pathways. Our gene-gene interaction results support this idea that the best model for BD indeed requires the interactions among *RORA, RORB*, and *NRID1* genes (Table 4). Thus, the *NR1D1* gene may exert its effect on BD through interacting with *RORs* gene family.

We reported associations of several genetic variants in *RORA* gene with BD in the present study. In a genetic association study with 19 circadian genes in the Spanish population, Soria et al. [21] reported that nine circadian genes (*ADCYAP1, ARNTL, ARNTL2, CLOCK, CRY1, NPAS2, PER3, VIP*, and *VIPR2*) exhibited nominal significance for major depressive disorder (MDD), whereas 3 genes (*BHLHB3, CSNK1E*, and *RORA*) were significantly associated with BD. This finding is consistent with ours that *RORA* demonstrates a significant association with BD in gene-based testing (P = 0.0007). While *RORA* gene did not show association with MDD in the Spanish samples, positive associations were reported in Finland samples for MDD and the presence of early morning awakening and fatigue [12]. These results may suggest the non-specific roles of *RORA* gene in mood- and sleep-related phenotypes, but not specific to BD diagnosis.

For the *RORB* gene, marker rs1327836 showed association with BD (OR = 1.75, P = 0.003) in the present study. Two prior studies also examined the *RORB* gene with BD (Table 1) and reported associations with different markers in this gene. Mansour et al. [20] studied 21 circadian genes and reported that the *RORB* gene was associated with both BD subtype I and schizophrenia. Another study found that four SNPs in *RORB* gene (rs1157358, rs7022435, rs3750420, and rs3903529) demonstrated associations with pediatric BD in Caucasian samples [19]. We noted that our significant marker was also genotyped in McGrath et al.’s [19] study without significant finding; the MAF of rs1327836 was much higher in our BD samples (0.37) than that in Caucasians (0.12). On the other hand, their reported significant markers did not show association with our BD samples either. Although allelic heterogeneity may explain these observations, it is also possible that associated markers reported in these studies merely tagged to other untested genetic variants in the *RORB* gene. Our significant marker, rs1327836 is located in the intronic region of the gene, which does not code for amino acids. Nevertheless, it is still possible that intronic markers influence eukaryotic gene expression through different regulation mechanisms, such as miRNA or methylation [32].

No markers in *NR1D1* showed main effects with BD in our samples. Results from previous genetic association studies (summarized in Table 1) are inconsistent with predominantly negative findings. Four out of five studies did not reported significant associations for *NR1D1* with BD. In accordance with our negative findings, this may imply that *NR1D1* does not play a major role in the pathophysiology of BD but interact with other genes to exert its effect. Moreover, it is found that lithium, a popular treatment for manic episodes [33], can inhibit glycogen synthase kinase 3β (*GSK3β*) and facilitate the degradation of *NR1D1* [34]. Therefore, *NR1D1* may not have direct effect on diagnosis *per se*, but influences on treatment response in BD. For instance, rs2314339 in *NR1D1* is reported to be associated with lithium response [35]. It might be interesting to study the effect of *NR1D1*gene with other phenotypes in our BD patients in the future, such as lithium treatment response.

For complex psychiatric disorders, the effect of a single genetic variant can be relatively small and it is assumed that multiple loci would exert their effects jointly or interactively in the pathogenesis of BD. For instance, Shi et al., (2008) conducted an association study of 15 circadian genes in BD family. They found significant multi-locus interactions between *BHLHB2, CSNK1E*, and *CLOCK* genes [47]. In the current study, the non-parametric MDR approach allows for interactions testing without the main effects. Combinations of several markers were identified by MDR to be associated with increased risk of BD, including rs2071427 in *NR1D1*, rs4774388 in *RORA*, and rs3750420 and rs11144047 in *RORB*. In the biological loops in circadian rhymes, *NR1D1, RORA*, and *RORB* completed ROREs binding in *ARNTL*. Ueda et al., [36] found that the NR1D1/ROR response element play an important role in generating circadian night expression in phase with *ARNTL* gene expression *in vitro* validation of clock-controlled elements. Thus, the possible mechanisms contributing to such joint actions may include the *ARNTL* gene and other related circadian genes, which additively or synergistically contribute to increasing the risk for BD. Our findings of potential interaction effects among these genes revealed supporting evidence for the associations between *NR1D* and *ROR* families and bipolar illness. Further studies can be expanded to explore all possible interacting effects in circadian pathways for BD. We also noticed that several previous GWA studies of BD have identified few susceptible loci for BD, such as *DGKH* (diacylglycerol kinase eta), *MYO5B* (myosin 5B), *ANK3* (ankyrin G), *SP8*, etc [37–41]. Although none of the typical circadian genes reached genome-wide significance level, one GWA study [42] reported that *BHLHB3* (a circadian pathway gene) exhibited moderate effect (OR = 1.26, p-value = 5.9×10^–5^) with BD. Recently, a convergent functional genomics approach is proposed to mine genetic signals from GWAS datasets as well as other resources and individual studies [43]. Interestingly, a few genes in the circadian pathway are ranked on the top for their associations with BD, such as *RORA, RORB*, and *ARNTL* genes.

A number of studies have established a close link between nuclear receptor function and expression, circadian regulatory circuitry, and regulation of various physiological processes [14, 44]. Owing to *RORA* and *RORB* function as nuclear receptors [45], they exhibit action in core circadian clock genes and in metabolic genes downstream. An animal model of BD reported altered expressions of *RORA* and *RORB* in the amygdala and pre-frontal cortex [46]. It is worthwhile to investigate the molecular basis underlying bipolar illness through understanding the functions of the encoded proteins in the circadian pathways.

In conclusion, our results support the involvement of genes in the *ROR* family in the pathogenesis of bipolar illness. Genetic polymorphisms in the *RORA* and *RORB* genes may confer risk for developing BD. The circadian pathway may be an important candidate for further evaluation in studying the pathogenesis of BD. More replication studies and basic research are needed to investigate the functional properties of these genes, and will contribute on our understanding of the etiological mechanisms underlying bipolar illness.

### Limitations

There are several limitations that should be noted. First, the power to detect genes with small effect sizes may be low due to the relatively small number of samples in this study. In addition, we did not formally correct for multiple testing as the selected markers may have higher *priori* to be associated with BD, and using the Bonferroni correction would thus be too stringent. However, to obtain empirical p-values, we applied permutation tests that assume the association effects are under the null. Although permutation tests cannot completely guard against inflated type-I errors, we sought for converging evidence using two samples to investigate the associations between circadian pathway genes and BD. Despite using a GWA chip with a dense marker set, the genotyped markers in the Affymetrix array are not evenly distributed in the genome, and there is no guaranteed that the coverage of variations in the three genes is high enough to capture haplotypic variations in tested genes. In addition, due to small sample size, we only included markers with a MAF >0.05, and common SNPs usually have small effects. The current study is not able to identify rare SNPs with large effects, untyped SNPs, and some structural variations, such as microsatellites, variable number tandem repeats, insertions, deletions, and duplications in the three genes for their associations with BD. Third, some of the associated markers are intronic markers. Further sequencing and functional studies are required to confirm whether the disease associations of reported markers are causal.
